# Lessons from export to New Zealand of the second opinion appointed doctor scheme

**DOI:** 10.1192/pb.bp.113.046540

**Published:** 2015-04

**Authors:** John Dawson, Paul Glue, Pete M. Ellis, Jessie Lenagh-Glue, David Goldsmith, Don A. R. Smith

**Affiliations:** 1 University of Otago, Dunedin; 2 University of Otago, Wellington, New Zealand; 3 Waitemata District Health Board, Auckland

## Abstract

**Aims and method** We compared findings of an audit of New Zealand’s version of the second opinion appointed doctor (SOAD) scheme with published information on the equivalent scheme for England and Wales, to consider what might be learnt from the different jurisdictions’ experience.

**Results** Strong similarities exist between the two schemes in the demographic profile of individuals subject to the SOAD process and rates of approval of compulsory treatment. The clearer legal framework for the English scheme and its supervision by an independent national agency may offer significant advantages in terms of consistency and transparency, compared with the informal, decentralised structure of New Zealand’s scheme.

**Clinical implications** Clinicians may not always favour greater formality or elaborate national structures for administering the Mental Health Act, but there are advantages in promoting clarity and consistency in a mandatory statutory process designed to protect compulsory patients’ rights.

In 1992, New Zealand adopted a modified version of the second opinion appointed doctor (SOAD) scheme into its mental health law. That scheme was first enacted for England (and Wales) by the Mental Health Act 1983 (UK). As in England, New Zealand law requires the proposals of the treating clinician to be approved by a second psychiatrist in two main situations – for longer-term use of medication, and for electroconvulsive therapy (ECT) – where a compulsory patient does not consent.^1^ In England, this mandatory second opinion scheme has been managed, funded and periodically reviewed by a national agency, firstly by the Mental Health Act Commission (MHAC), then by the Care Quality Commission (CQC). In New Zealand, no equivalent national agency has existed to manage the scheme. Its administration has fallen on regional officials (usually senior psychiatrists) who manage the statutory process in the nation’s 20 district health boards. Moreover, New Zealand’s national guidelines on the Mental Health Act^2,3^ contain nothing like the detail of the English Code of Practice^4^ regarding conduct of the scheme. Instead, each local district health board uses its own systems and forms.

The English rules require SOADs to check the patient’s legal status, speak to the patient in private, consult the treating clinician, consult two other professionals involved in the patient’s care and provide written reasons for the decision. A national system of online forms is also used for SOADs to document these steps, state whether they approve the treatment in changed or unchanged form and specify the agreed treatment regime.^5^ No such prescriptive rules govern the New Zealand process. This paper compares the role performed by second opinion psychiatrists in New Zealand with that of SOADs in England and Wales. The term SOADs will be used to refer to the second opinion psychiatrists, of both jurisdictions.

## Method

We audited the New Zealand scheme, for the first time, at three different centres. We reviewed documentation concerning SOADs’ conduct of the treatment approval process, for both medication and ECT.^6^ We matched SOAD reviews of ECT with the two medication reviews nearest in time (ECT, *n* = 146; medication, *n* = 292; total, *n* = 438). Then we studied the progress in the following year of 11 patients at one centre whose treatment had not been fully approved by the SOAD.^7^ Finally, we compared our findings with published information and guidelines concerning the equivalent English scheme. The study was approved by New Zealand’s Multi-Region Ethics Committee.

## Results

### Comparisons between the New Zealand and English SOAD schemes

We found intriguing similarities and important differences between the operation of the New Zealand and English schemes. There were strong similarities in the demographic profile of patients for whom treatment approval was sought for medication and ECT respectively. In both jurisdictions, in medication reviews, males outnumbered females by approximately 2:1, whereas that gender ratio was reversed for ECT; the mean age of patients undergoing medication review was significantly younger than for ECT; and the mean age of female patients under review was significantly older than males, for both medication and ECT.

These features seem to be associated with the different diagnostic profile of patients undergoing the different forms of treatment. In New Zealand, we found patients undergoing medication review tended to be male (62%), younger (mean age 44.8 years) and had a diagnosis of schizophrenia or schizoaffective disorder (64%), whereas patients being considered for ECT tended to be female (70%), older (mean age 56.9 years) and had a diagnosis of affective disorder (68%).^6^ Interestingly, Māori patients tended to be underrepresented in figures for ECT (6%) compared with their proportion in the general New Zealand population (approximately 15%).

In both jurisdictions, ECT reviews generally occur at an early stage in the Mental Health Act process. In England, Fennell, in a study conducted some years ago, found 60% of ECT approvals occurred within 7 days of the patient’s detention under the Act, 18% on the very first day.^8^ In New Zealand, we found 60% occurred during the initial month’s compulsory assessment under the Act.^6^ Medication reviews are only required by the legislation, of both jurisdictions, after the patient has been detained for a longer period of time.

**Fig. 1:**
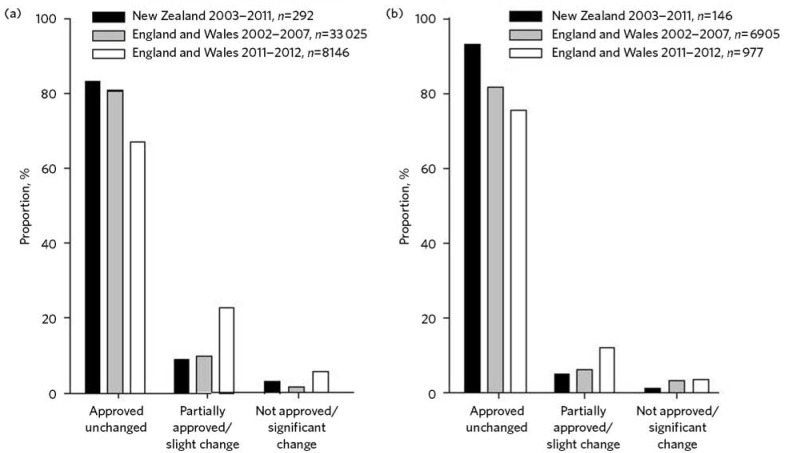
Extent of approval of (a) medication and (b) electroconvulsive therapy (ECT) by second opinion appointed doctors (SOADs) in New Zealand (2003–2011) and England and Wales (2002-2012). Data from Dawson *et al* (2013),^6^ Mental Health Act Commission (2008),^9^ Care Quality Commission (2013).^11^ Terms used in New Zealand: ‘approved unchanged’, ‘partially approved’ and ‘not approved’. Terms used in England and Wales for both periods of time: ‘approved unchanged’, ‘slight change’ and ‘significant change’. There were missing data for second opinions on medication (New Zealand 2003–2011, 2.4%, England and Wales 2002–2007, 7.6% and 2011–2012, 3.5%) and ECT (New Zealand 2003–2011, 0%, England and Wales 2002–2007, 8.2% and 2011–2012, 8.3%).

In both jurisdictions, SOADs overwhelmingly approved the treatment sought, especially ECT. Non-approval, or significant change in the proposed treatment, was rare (Fig. 1).^6,9–11^ In England, there has been a recent trend towards less frequent full (or ‘unchanged’) approval of treatment plans for medication (81% in 2002–2007, falling to 68% in 2011–2012), though not for ECT.^9,11^

### Lack of consistency between New Zealand centres

In our New Zealand audit, we found little consistency in the conduct of the SOAD process at the different centres.^6^ There were marked differences in how SOADs were designated to review the treatment of individual patients; the number of clinicians who performed the SOAD role; their degree of independence from treating clinicians; the forms they completed; and the steps they took during the approval process, as documented in local forms or the patient’s clinical record in the week before or after treatment was reviewed.

In New Zealand, the Mental Health Act authorises the Mental Health Review Tribunal to appoint qualified psychiatrists as SOADs, but there is no national agency managing the scheme.^1^ Different methods are then used in the various regional district health boards to designate the particular SOAD who will review an individual patient’s treatment. These methods include an administrator approaching SOADs on a roster system; the treating clinician sending an email request to all local SOADs, to see who responds; and the treating clinician directly approaching a SOAD with specialised knowledge in treating the particular patient’s condition. In some services, treating clinicians personally decide which SOAD to approach.

At one centre, only a small number of appointed SOADs actually performed the task. At another, the work was shared widely among consultant psychiatrists. At a third, a single specialist considered almost all proposals for approval of ECT, then administered the course of treatment, if approved. The SOADs invariably worked in the same region as the treating clinician. They were not paid more to perform the SOAD role than their usual salary or given any particular relief from their usual workload.

**Fig. 2:**
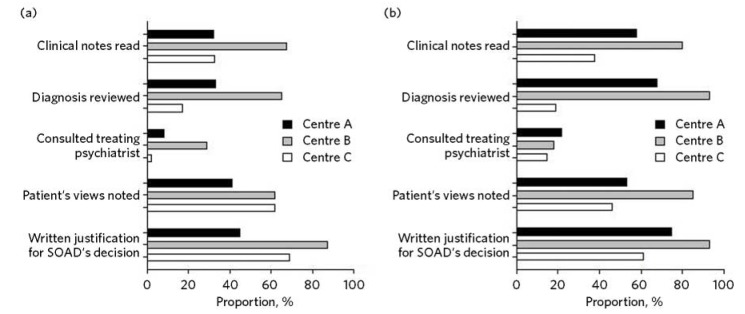
Recorded elements at three New Zealand centres in second opinion appointed doctor (SOAD) reviews of (a) medication and (b) electroconvulsive therapy (ECT).

Each district health board used different forms and systems for recording the SOADs’ written opinions on treatment. The text of these opinions was remarkable in its diversity, ranging from a single word (the name of an alternative medication) to a three-page formal report. The depth of scrutiny given by SOADs to the treatment is indicated in part by this written record. It revealed great variation between New Zealand centres in the extent to which SOADs recorded having reviewed the patient’s clinical notes, diagnosis or treatment plan, or recorded the patient’s views on treatment. There was similar variation in the extent to which there was any record that SOADs had spoken to the treating clinician or provided a written

justification for their decision. This diversity is illustrated in Fig. 2.

In short, there seems little consistency in SOADs’ conduct of the process at different New Zealand centres. Moreover, different methods were used to store information generated during the process, and generally no adequate method was adopted for linking the information SOADs provided on the forms with any comments they made in the patient’s notes, so the two could be read together.

### No clear process where impasse

At one centre we evaluated the files of 11 patients whose treatment had not been approved, or only partially approved, by the SOAD,^7^ trying to determine how the disagreement had been resolved. We found that when the SOAD did not approve, or qualified, the treatment plan, intensive consultation usually occurred between the treating clinician, the SOAD and the regional administrator of the Act. Cases generating most correspondence concerned non-approval of ECT. In some cases, these written exchanges revealed significant dissatisfaction or disagreement on the part of the clinicians.

In England, the Mental Health Act Code of Practice says (para 24.67) there is ‘no appeal’ from the SOAD’s decision to approve treatment or not.^4^ In New Zealand, the Act simply says that, for the proposed treatment to proceed, it must be approved by ‘a’ SOAD. This rule does not say the SOAD can veto the treatment proposed. Instead, it opens the possibility that another SOAD might approve the treatment, if the first SOAD does not. So it might be said that there is an appeal.

In 2 (of 11) cases of non-approval we followed, further second opinions were sought when the first SOAD declined. Nevertheless, some disagreement arose in these cases between the clinicians as to whether the initial SOAD’s refusal to approve treatment was final. No clearly established process seemed to exist for resolving such disagreements, and no clear ‘appeal’ process was specified by the Mental Health Act guidelines^2^ during the period studied.

## Discussion

### Main findings

Our findings show some clear similarities in the conduct of the SOAD schemes in England and New Zealand, although there is considerable inconsistency in the process followed at different New Zealand centres, along with uncertainties arising from the absence of a clear rule in New Zealand regarding the finality of a SOAD’s decision not to approve treatment.

There are similarities in the characteristics of patients subject to medication and ECT reviews, in the stage patients have reached in the civil commitment process when undergoing these reviews and in the high rates at which SOADs approve the treating clinicians’ plans. In sum, under the two schemes, SOADs seem to review similar patients, at similar stages in the Mental Health Act process, with similar results.

One can debate whether high rates of approval of treatment by SOADs are a good or a bad thing. Psychiatrists exercise considerable discretion in selecting appropriate treatment and, in doing so, must take many factors into

account. Complete agreement between treating clinicians and SOADs on all occasions is therefore improbable. If it occurred, it would suggest ‘rubber-stamping’ and no exercise of independent judgement on the part of SOADs. That would seem to rob the process of any value. Yet high levels of disagreement between the two clinicians would also be a concern. It would seem to call into question the competence of SOADs, or the competence of treating clinicians whose plans would be regularly overruled. Alternatively, it would call into question the reliability of treatment decisions in psychiatry, if the two clinicians could rarely agree on a treatment plan.

A high but not complete level of agreement therefore seems satisfactory. It suggests that independent judgement is exercised by SOADs, but the two clinicians can usually agree on a treatment plan. The rates of approval, in both New Zealand and England, fit that pattern, even though the rates are not exactly the same.

### Lessons learnt

At the New Zealand centres studied, there were notable inconsistencies in how SOADs were designated to review individual patients’ treatment and in the degree of independence evident between SOADs and treating clinicians. There also seemed to be little consistency in the range of people SOADs consulted or in the information they considered (including the frequency with which they consulted the treating clinician, considered the patient’s views about treatment and provided a written justification for their decision) – as judged from their written comments on the forms or in patients’ notes. Likely explanations for this diversity include the lack of detailed national guidelines stipulating a process to follow, lack of specific training or funding for SOADs who perform the role, and absence of decisions of New Zealand courts reviewing the SOADs’ conduct against legal standards of procedural fairness.

A notable feature of the New Zealand situation is the absence of a ‘no appeal’ rule. In cases where SOADs declined to approve treatment, intense consultation usually occurred, but this did not always produce agreement between the SOAD and treating clinician on an amended treatment plan. Approval from another SOAD might then be sought, on the premise that treatment could proceed if ‘a’ positive opinion was obtained.

The ‘no appeal’ rule in England is more final and certain. It may encourage SOADs and treating clinicians to negotiate an amended treatment plan to permit some form of agreed treatment to proceed, when the patient is detained for treatment under the Act. It is interesting that the MHAC and CQC publish no figures on cases in which SOAD approval is declined. They report only cases in which the treating clinician’s plan was approved ‘unchanged’, ‘slightly changed’, or ‘significantly changed’. There is in fact no space on the current CQC form for English SOADs to say they decline. Presumably they could simply decline to sign the form and – if there was ‘no appeal’ – that would seem to mean the treatment could not proceed.

This does not mean that all disagreements in England between treating clinicians and SOADs are happily resolved. The ‘no appeal’ rule may confer sufficient authority on SOADs to secure amendment of the treatment plan, in most cases, and conferring such authority on SOADs may be more readily justified where – as in England, but not New Zealand – senior psychiatrists are selected, trained, funded and supervised by an independent national agency to perform the task. But it is not wholly obvious why one SOAD should have final authority to approve the treatment or not, and some treating clinicians are no doubt left aggrieved by the SOAD’s decision. The SOAD does not carry continuing responsibility for the patient’s care, and the treating clinician may have far more knowledge of the patient and be a specialist in treatment of the patient’s particular condition. So why should the treating clinician be overruled by another clinician, with no right of appeal?

Greater knowledge on the part of the treating clinician should, of course, be taken into account by the SOAD when making their decision. But a case can be made for the New Zealand position: that treatment should be permitted provided ‘a’ SOAD approves. This produces something like an appeal from the first SOAD’s decision. Any appeal process should be clearly specified, however, and should not be capable of manipulation by the treating clinician.

The new New Zealand guidelines regarding the Mental Health Act, issued in 2012^3^ after closure of the period we studied, address the matter more fully. They suggest (at para 10.2.2) that, where the first SOAD declines to approve, the regional administrator of the Mental Health Act (although not the treating clinician) may ‘direct that another approved psychiatrist provide a second opinion’. So the process of obtaining another SOAD’s opinion is to be managed by a senior psychiatric administrator, not by the treating clinician. One can imagine such a process being managed in England by the CQC.

Nevertheless, through this ‘appeal’ process the first SOAD’s view can be trumped. So then we may ask: why should the second SOAD’s view be preferred to the first, and will ‘shopping around’ for opinions somehow occur? In the end one might conclude that the practical advantages of finality justify accepting the first SOAD’s view, and support the ‘no appeal’ rule.

Other changes to the New Zealand scheme might be made to try to capture some advantages of the English superstructure. The new guidelines in New Zealand suggest SOADs should consider, before approving treatment: the history of the patient’s illness and prior pharmaceutical regime; the risks and benefits of potential treatment approaches; the patient’s views, as far as they can be ascertained; and whether the treatment is of maximal benefit to the patient and appropriate to their condition.^3^ This provides some guidance on the process to follow. But the guidelines could go further, to specify clearly the degree of independence required between SOADs and treating clinicians, and the information SOADs should record. The Ministry could promulgate a system of online forms to be used nationally. Completion of the forms would confirm the necessary steps have been taken, and the forms could be used to collate data, publish statistics and make the process more transparent, as has occurred in England with reports from the MHAC and CQC.^9–11^

### Limitations

The shortcomings of this study must be acknowledged. The retrospective data collection for our audit was based on written forms and clinical notes. It is a study of documented steps and is likely to underestimate the intensity of treatment review SOADs conducted. Our general audit only covered the process at 3 New Zealand district health boards (out of 20) and our substudy covered a small number of non-approved cases at a single board. The audit discovered significant diversity in practice between district health boards, so generalising to other boards may be inappropriate.

### Implications

In our audit, 438 examples of the SOAD process were studied at the three sites. These related to both medication and ECT, and the results show clear parallels between the operation of the New Zealand and English schemes. Our substudy of non-approved cases is, we believe, the first of its kind. In conclusion, the inconsistencies revealed in the conduct of the process at different New Zealand centres should encourage clinicians in England and Wales to value their clear national guidelines and forms. It shows the benefits of the structure, training, funding and reporting provided by the MHAC and CQC. Clinicians may not always value greater formality or elaborate structures for administration of the Mental Health Act. But, in light of New Zealand’s experience, we suggest that central administration of a SOAD scheme can confer advantages in terms of clarity and consistency that are particularly desirable in a mandatory process designed to protect compulsory patients’ rights.

## References

[1] New Zealand Government. Mental Health (Compulsory Assessment and Treatment) Act 1992 (NZ), sections 59, 60. New Zealand Government, 1992.

[2] New Zealand Ministry of Health. Guidelines to the Mental Health (Compulsory Assessment and Treatment) Act 1992. Ministry of Health, 2000.

[3] New Zealand Ministry of Health. Guidelines to the Mental Health (Compulsory Assessment and Treatment) Act 1992. Ministry of Health, 2012.

[4] Department of Health. Code of Practice – Mental Health Act 1983. TSO, 2008.

[5] Care Quality Commission. SOAD Report Form. Care Quality Commission, 2013 (www.cqc.org.uk/mha).

[6] Dawson J Ellis P Glue P Lenagh-Glue J Goldsmith D Smith DA, Mandatory second opinions on compulsory treatment. In New Zealand’s Mental Health Act in Practice (ed Dawson J Gledhill K): 229–46. Victoria University Press, 2013.

[7] Lenagh-Glue J Glue P Dawson J When the mandatory second opinion fails to approve compulsory treatment. Australasian Psychiatry (in press). 10.1177/1039856214537879 24899510

[8] Fennell P Treatment Without Consent. Routledge, 1993.

[9] Mental Health Act Commission. Risk, Rights, Recovery: 12th Biennial Report 2005-2007. TSO, 2008.

[10] Mental Health Act Commission. Coercion and Consent: 13th Biennial Report 2007-2009. TSO, 2009.

[11] Care Quality Commission. Monitoring the Mental Health Act in 2011/12. CQC, 2013.

